# A Unique Panel of Patient-Derived Cutaneous Squamous Cell Carcinoma Cell Lines Provides a Preclinical Pathway for Therapeutic Testing

**DOI:** 10.3390/ijms20143428

**Published:** 2019-07-12

**Authors:** Sakinah Hassan, Karin J. Purdie, Jun Wang, Catherine A. Harwood, Charlotte M. Proby, Celine Pourreyron, Nikol Mladkova, Ai Nagano, Sandeep Dhayade, Dimitris Athineos, Matthew Caley, Viviana Mannella, Karen Blyth, Gareth J. Inman, Irene M. Leigh

**Affiliations:** 1 Blizard Institute, Barts and the London School of Medicine and Dentistry, QMUL, London E1 2AT, UK; 2 Barts Cancer Institute, QMUL, London EC1M 6BQ, UK; 3 Division of Cancer, Ninewells Hospital and Medical School, University of Dundee, Dundee DD1 9SY, UK; 4 Cancer Research UK Beatson Institute, Garscube Estate, Switchback Rd, Glasgow G61 1BD, UK; 5 Institute of Cancer Sciences, University of Glasgow, Glasgow G61 1GH, UK

**Keywords:** squamous cell carcinoma, cutaneous, in vitro, keratinocytes

## Abstract

Background: Cutaneous squamous cell carcinoma (cSCC) incidence continues to rise with increasing morbidity and mortality, with limited treatment options for advanced disease. Future improvements in targeted therapy will rely on advances in genomic/transcriptomic understanding and the use of model systems for basic research. We describe here the panel of 16 primary and metastatic cSCC cell lines developed and characterised over the past three decades in our laboratory in order to provide such a resource for future preclinical research and drug screening. Methods: Primary keratinocytes were isolated from cSCC tumours and metastases, and cell lines were established. These were characterised using short tandem repeat (STR) profiling and genotyped by whole exome sequencing. Multiple in vitro assays were performed to document their morphology, growth characteristics, migration and invasion characteristics, and in vivo xenograft growth. Results: STR profiles of the cSCC lines allow the confirmation of their unique identity. Phylogenetic trees derived from exome sequence analysis of the matched primary and metastatic lines provide insight into the genetic basis of disease progression. The results of in vivo and in vitro analyses allow researchers to select suitable cell lines for specific experimentation. Conclusions: There are few well-characterised cSCC lines available for widespread preclinical experimentation and drug screening. The described cSCC cell line panel provides a critical tool for in vitro and in vivo experimentation.

## 1. Introduction

Cutaneous squamous cell carcinoma (cSCC) is a common and growing problem, with over 45,000 cases per year in the UK in 2015 [1]. There is a dearth of effective treatment for high-risk and metastatic disease, which has a high mortality (40% within 3 years), but the advent of immunotherapy for melanoma has opened up the possibility of treating advanced cSCC with PD1/PD-L1 and CTLA4 targeted immunotherapies [2]. However, targeted therapy remains elusive; cSCC has a very high mutational burden with the commonest mutated genes being Notch 1 and 2, TP53 and CDKN2A [3,4]. Although the application of new genomics and transcriptomics has been informative in primary invasive cSCC and precursor lesions, the genetic drivers of metastasis are poorly understood [5]. Testing the functional effects of putative genetic drivers requires robust preclinical testing, as do the high throughput screening platforms for drug discovery. A considerable number of keratinocyte cancer cell lines from head and neck SCC (HNSCC) have been developed over the years [6]. They have been widely used in in vivo and in vitro modelling to study the biology of HNSCC, although genotyping has revealed the extent of cross-contamination and mistaken identity of these lines (37/85) [6]. In comparison, few cSCC cell lines have been developed. We report a panel of 16 patient-derived cSCC lines derived in our laboratory. We clarify and define the nomenclature for these lines, referencing also their use in previous publications. We describe their genetic characterisation and show that they closely recapitulate the biology of primary tumours, both in vitro in 2D and 3D culture, and also in vivo in subcutaneous xenografts, providing a unique resource for preclinical testing.

## 2. Results

### 2.1. Case Histories and Histopathology

**Figure 1:**
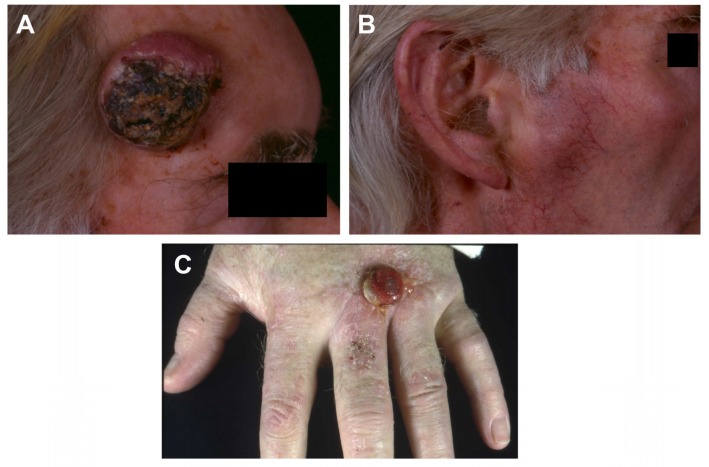
Clinical presentation. Primary cutaneous squamous cell carcinoma (cSCC) on the temple of an immunocompetent male which gave rise to the IC1 cell line (**A**). Lymph node metastasis which gave rise to the IC1MET cell line (**B**). Local recurrent cSCC on the hand of an immunosuppressed male transplant recipient, giving rise to the MET2 cell line (**C**).

**Figure 2:**
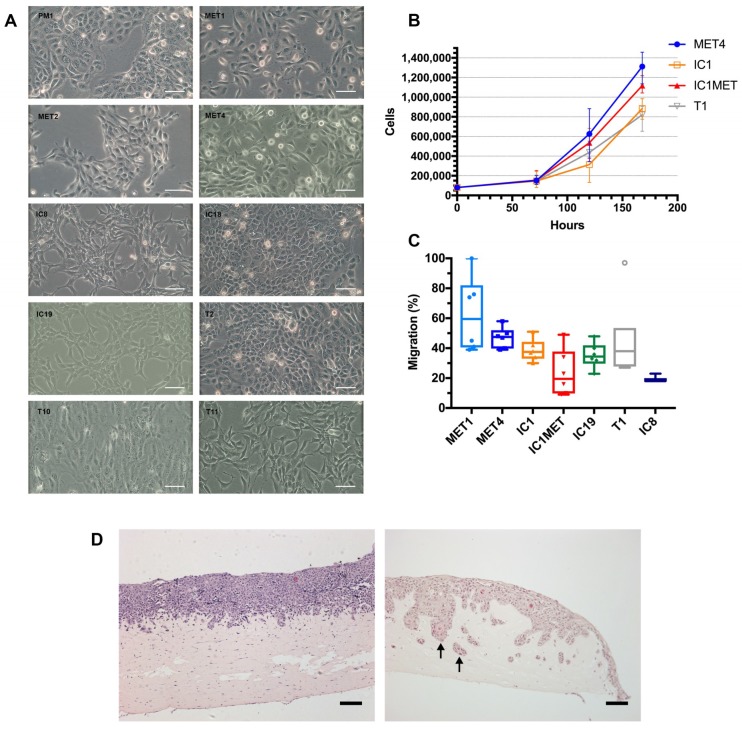
cSCC cell line characterisation. Phase contrast images demonstrate that the cSCC cell lines have distinct morphologies based on the differentiation status of the tumours from which they were derived and their metastatic potential (**A**). The growth rate of two metastatic, MET4 and IC1MET, and two primary, IC1 and T1, cSCC lines; two of which were isogenic lines, IC1MET/IC1 (**B**). Transwell migratory potential of a selection of the cSCC lines, including four immunocompetent patient-derived and three immunosuppressed transplant recipient-derived lines; representing two separate isogenic line pairs, IC1/IC1MET and MET1/MET4, alongside three primary lines (**C**). Organotypic hematoxylin and eosin (H&E) images of two immunocompetent cSCC lines, IC8 and IC1. IC1 was derived from a tumour that went on to metastasise. Invading cells are shown by arrows (**D**). Scale bars = 100 µm. Values presented as mean ± SEM. Experiments performed twice in triplicate (**A**–**C**) and a total of three times (**D**).

**Figure 3:**
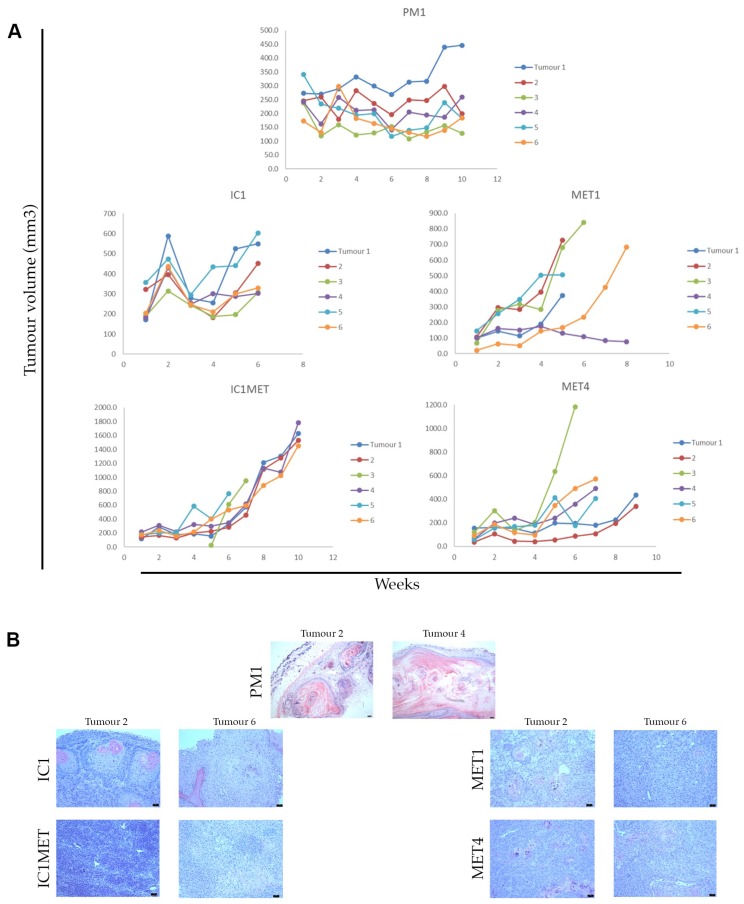
Subcutaneous xenografts. Summary of the tumour growth kinetics of the indicated cell lines in subcutaneous xenografts (*n* = 6 per cell line) (**A**). H&E staining of the representative sections of the indicated xenografts harvested at endpoint (**B**), scale bars = 100 µm.

**Figure 4:**
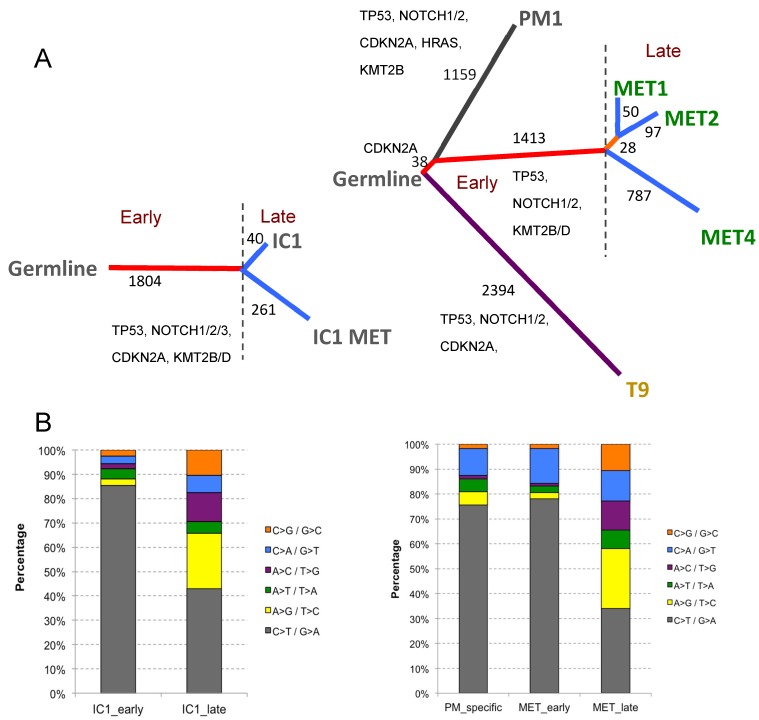
Phylogenetic analysis and mutational signatures of two isogenic cell line series. The numbers of non-synonymous truncal and branch mutations are indicated (**A**). A significant (*p* < 0.0001) decrease in C > T transitions accompanied by a significant (*p* < 0.0001) increase in A > G transitions was observed during the evolution of both tumour series (**B**). IC1/IC1MET, paired primary and metastatic cSCC from an immunocompetent individual; MET1/MET2/MET4, cell lines derived from a primary cSCC and its recurrence and metastasis, respectively, from an immunosuppressed organ transplant recipient; PM1, premalignant cell line generated from dysplastic skin from the same patient; T9, cell line generated from a distinct primary cSCC from the same patient.

A total of 16 cell lines were derived from 11 patients with cSCC attending dermatology outpatient clinics. Five of these 11 patients were immunocompetent individuals (three male and two female) and six of these 11 patients were immunosuppressed male organ transplant recipients (OTR) aged 51–87 years and 45–67 years, respectively, at the time of biopsy (Table 1). One cell line was derived from premalignant forehead skin (PM1), 13 of the 16 cell lines were derived from primary cSCC lesions and two from lymph node metastases. One of the immunosuppressed donors contributed five of the 16 cell lines: one premalignant, three primary and one metastatic cSCC [7] (Figure 1). One of the immunocompetent donors (77-year-old male) contributed two lines (IC1 and IC1MET), derived from a moderately differentiated primary cSCC arising on the right temple and its subsequent metastasis to the right pre-auricular lymph node (Figure 1). The clinical details, histological classification (from well- to poorly-differentiated) and immunosuppressive drug details are shown in Table 1. Some of the cell lines have been developed over the last 30 years [7] but the majority over the last 10 years. There has been remarkable conservation of findings following passaging. All lines were established by the same technique as described in the methods section. However, before performing whole exome sequencing (WES), cell lines were re-derived and full characterisation, including WES, was performed on an established collection. This collection is now being made widely available, and the publication refers to this collection only and not to any earlier derivations of the cell lines. The only observed difference between immunosuppressed and immunocompetent cell lines was the finding of an azathioprine signature on whole exome sequencing in lines from immunosuppressed patients [3]. None of the other functional observations, both described in this and previous papers, have segregated cell lines according to the immune status of the donor (Figure 2, Figure 3, Figure 4 and Table A1).

### 2.2. In Vitro Characteristics: Cell Morphology, Growth Curves, Transwell Migration and Organotypic Invasion

Cell morphology representative of nine of the 16 lines is shown in Figure 2A. Cells from two primary, IC1 and T1, and two metastatic lines, MET4 and IC1MET, were seeded into six multiwell plates and growth measured at days 3, 5 and 7 (Figure 2B). All four lines entered into an exponential growth phase by 3–5 days in culture. Metastatic cells grew faster than primary cells, irrespective of the immune or differentiation status of the tumour from which the cells were derived. These four lines, along with cells from two additional primary lines (MET1 and IC19) were treated with mitomycin C, to ensure that migration was proliferation-independent, before being seeded into the upper chamber of transwell inserts and migrated for 18 h (Figure 2C). A greater proportion of cells from primary lines belonging to the isogenic cell line pairs (MET1/MET4 and IC1/IC1MET), migrated compared to their isogenic metastatic counterparts. The remaining primary cell lines IC19 and T1 migrated to a lesser extent than MET1 but a similar extent to IC1. Cells from the primary lines IC8 and IC1 were seeded onto collagen/matrigel matrices containing normal human fibroblasts and grown for 7 days at 37 °C (Figure 2D). Both lines formed epidermal layers, with IC1 showing greater levels of invading cells that invaded deeper into the dermis compared to IC8. The morphology of the IC1 keratinocytes shows dysplastic features.

### 2.3. In Vivo Tumorigenicity

Fourteen of the 16 cell lines were investigated in xenograft models. The occurrence and growth rate of tumours developing after subcutaneous injection varied between cell lines (Table 1, Figure 3A). Palpable tumours developed in 100% of severe combined immune deficiency (SCID) mice injected with IC1, IC1MET, MET1, MET4, PM1 within the first 2 weeks of injection, with some intra-tumoural growth variability observed. PM1 cells established tumours which maintained a stable volume for 10 weeks when they were harvested (Figure 3A). MET1 and MET4 tumours grew with some variability, and several tumours were harvested upon ulceration. IC1 tumours grew rapidly but some regression was seen between 2–4 weeks post-injection, prior to further tumour growth and then variable subsequent regression after 6 weeks (Figure 3A and data not shown)—suggesting that IC1 xenografts could be a suitable choice as short-term drug testing models. IC1MET tumours grew with consistent kinetics and could be a suitable model for longer-term drug testing. The histology of the xenograft recapitulated the pathology of the original tumours, with ulceration apparent in PM1 xenografts (Figure 3B).

### 2.4. Genetic Analyses

#### 2.4.1. Short Tandem Repeat (STR) DNA Profiling

Fifteen STR loci plus amelogenin were genotyped to give tumour-specific profiles (Table 2). These profiles authenticated all cell lines as human and were unique to the patient of origin and distinct from previously published human cell line profiles in the American Type Culture Collection (ATCC) STR database. For the two isogenic line sets IC1/IC1MET and MET1/MET2/MET4, tumour recurrences or metastases demonstrated STR profiles identical to the primary tumour. In contrast, two isogenic lines (PM1 and T9) belonging to the latter isogenic set (MET series) but derived from separate primary lesion sites demonstrated highly similar but non-identical profiles which could be distinguished from the MET lines by their genotype at the CSF1PO, D3S1358 and TH01 loci. 

#### 2.4.2. The Genomic Landscape of Cell Lines

Whole exome sequencing (WES) was performed on the 15 lines, for which matched normal samples were available (GSE98780) [3]. Our results suggested that mutational profiles, e.g., somatic mutations and copy number alterations (CNA) of cSCC lines, were comparable with those of the patient tumour sample set, properly reflecting the complexity of the disease. Genomic abnormalities observed in cell lines include mutations in many reported cSCC drivers, such as TP53, NOTCH1/2, CDKN2A, HRAS, SF3B1 and PTEN, as well as novel drivers, including ATP1A1 and GRHL2 [3]. The molecular profiling of these lines provides a unique valuable resource for studying the functional significance of novel molecular events responsible for cSCC development and progression.

#### 2.4.3. Phylogenetic Analysis of Isogenic Lines

The non-silent mutations identified were used to construct evolutionary trees for the two isogenic cell line sets, primary cSCC IC1 and its metastasis IC1MET, as well as for the three related (MET1/MET2/MET4) and two unrelated lines (PM1/T9) derived from the single immunosuppressed transplant recipient male patient (Figure 4A). A majority (86% and 60% for the IC1 and MET trees, respectively) of all non-synonymous mutations identified in the related lines were truncal (early), reflecting the clonal similarity between metastatic and primary lines (Figure 4A). For late branch mutations, most of them occurred in the metastatic branch, not the primary specific lines (87% and 82% for the IC1 and MET trees, respectively). All mutations in the known drivers (TP53, NOTCH1/2 and CDKN2A) appeared to be truncal, highlighting their significance in initiating and establishing the dominant clones (Figure 4A). We next looked for genomic events that could potentially drive and/or contribute to metastatic progression. Based on the phylogenetic analysis, we identified 58 commonly mutated genes between IC1MET and MET4 metastasis-specific branches, which were not mutated in any of the primary lines. Gene-set enrichment analysis using Database for Annotation, Visualization and Integrated Discovery (DAVID) Bioinformatics resources revealed that gene ontology (GO) biological processes, such as the regulation of transcription (fold enrichment, FE = 2.43; false discovery rate (FDR) = 9.68 × 10^−4^), chromatin modification (FE = 6.58, FDR = 2.72 × 10^−2^), RNA splicing (FE = 6.35, FDR = 3.17 × 10^−2^), and the mRNA metabolic process (FE = 4.87, FDR = 0.09), were significantly overrepresented for these 58 genes, highlighting the potential involvement of these pathways and processes in driving or significantly contributing to cSCC metastasis.

We next investigated the mutational signatures (with respect to transitions and transversions) for early and late mutations, and aimed to identify any significant differences between them. As shown in Figure 4B, a significantly higher proportion of C > T/G > A transitions (often induced by UV damage) was observed in truncal mutations (80%) compared to their branch counterparts (40%, Fisher’s exact test, *p* < 0.0001). In contrast, the proportion of other mutations became less abundant. In particular, there was a >10-fold increase in A > G/T > C transitions during the tumour progression, representing more than 20% of all late mutations for both series (Figure 4B). This suggests that signatures 5, 12 and 16 (see https://cancer.sanger.ac.uk/cosmic/signatures), which often consist of A > G/T > C substitutions, became more dominant after the tumours are fully established and during the tumour progression. Although signature 7 (UV light exposure) remained the most dominant signature throughout, its influence became important after the full establishment and during the progression and metastatic stages.

#### 2.4.4. Genome-Wide Methylation Profiling of cSCC Cell Lines

**Figure 5:**
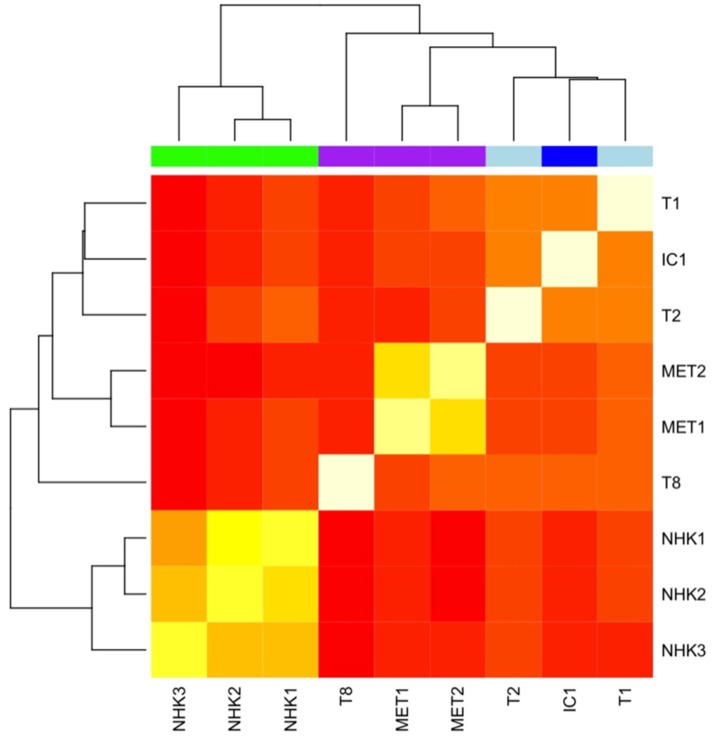
Heatmap of Pearson’s correlation of genome-wide methylation profiling of six cSCC cell lines and three normal human keratinocytes (NHK). NHK (green) cluster together separately from cSCC, poorly-differentiated cSCC (purple) form a cluster with the two isogenic cell lines—MET1 and MET2—forming a tight subcluster. Two well-differentiated cell lines (T1 and T2, light blue) formed a cluster with a moderately-differentiated cell line, IC1 (dark blue).

We then explored the methylation characteristics of six cSCC cell lines (T1, T2, IC1, T8, MET1, MET2) using genome-wide DNA methylation microarray. The cSCC lines were hybridised to the same chip with three normal human keratinocytes (NHK) to account for possible batch effects. Genome-wide methylation profiles reflected the original histologies (cSCC vs. NHK) and also differentiation status subtypes of cSCC based on Pearson’s correlation (Figure 5). Cell lines derived from poorly differentiated tumours formed a cluster, while cell lines derived from well- and moderately-differentiated cSCC (T1, T2, IC1) formed a separate cluster. A comparison of genome-wide methylation profiles of NHK and cSCC cell lines revealed a statistically significant difference in methylation in 361 unique genes (adjusted *p*-value ≤ 0.01), including known cancer drivers (MAP2K2, WNT5A, COL4A1, BMP2 and FGF7).

We then explored the potential enrichment of subsets of differentially methylated genes. Gene-set enrichment analysis revealed significant enrichment in several canonical pathways including MAPK, JAK-STAT, insulin signaling and the p53 pathway. P53 is known to play a critical role in cSCC oncogenesis, and genes involved in p53 signaling, IGF1, IGFBP3, CASP8, SERPINB5, were identified as being differentially methylated. This suggests that the genome-wide methylation profile retained by stable cell lines maintains the original tissue features and reflects the methylation changes in processes inherent to cSCC onset and progression.

## 3. Discussion

We describe for the first time a unique panel of 16 cSCC lines which have been extensively characterised in terms of clinical and histological phenotyping; genotyping by STR DNA profiling, exomic sequencing, copy number alteration analysis and phylogenetic analysis; in vitro cell morphology, growth, transwell migration, organotypic invasion; and in vivo tumourigenicity. This panel includes multiple isogenic lines and lines which represent the spectrum of tissue sites and histologies, and is a unique resource for preclinical cSCC research. Indeed, to date, a few of these lines have been used individually in multiple collaborative research efforts (Table A1).

### 3.1. Existing cSCC Cell Lines

There are few well-established cSCC cell lines. An online search using the cell line catalogue databases ATCC, the European Collection of Authenticated Cell Cultures (ECACC) and the German Collection of Microorganisms and Cell Cultures GmbH (DSMZ) revealed that the only SCC lines listed are of head and neck origin. As far as we are aware, the only cutaneous SCC line widely available commercially is A431. This line is of vulval origin [8,9,10] and, although there is no evidence of human papillomavirus (HPV) involvement, it is unlikely to reflect the mutational profile of UV-associated cSCC [3]. The panel of HNSCC lines developed by Rheinwald and Beckett in 1980 [11] included cell lines (SCC12, SCC13) which derived from a cutaneous SCC: clinical details of the site and tumour characteristics are not available, although these lines have been widely used experimentally [12]. There are a number of publications using the UT-SCC HNSCC lines, which include five primary cSCC lines (UT-SCC-12A, 91, 105, 111, 118) and three metastatic cSCC lines (UT-SCC-7, 59A, 115) [13,14,15], but the characterisation of these lines has not been reported comprehensively. SRB-1 and SRB-12 cell lines were derived from a moderately well-differentiated cSCC [6,16,17], and Colo16 was developed from a metastatic cSCC arising in a chronic burn scar on the leg of a 59-year-old black female [18]; all three have subsequently been used in drug discovery studies [19,20,21,22,23,24,25,26,27]. A model of skin carcinogenesis has been developed using mutant Ras/Cdk4 transfection of normal keratinocytes [28,29]; however, HRas mutations are uncommon in human cSCC. HaCaT cells were spontaneously immortalised from clinically normal peritumoural tissue and are widely used as immortalised keratinocytes as they closely resemble normal keratinocytes in their growth and differentiation potential [30]. The parent line is non-tumorigenic through multiple passages (320), but malignant subclones have been developed following transfection with mutated val-12 Harvey Ras oncogene [31]. Our panel therefore constitutes the largest panel of patient-derived cSCC cell lines and is the most comprehensively characterised.

### 3.2. Tumour Microenvironment

Cancer-derived cell lines lack the critical influence in vivo from the tumour microenvironment, but this can be mimicked in organotypical cultures and subcutaneous xenografts. We present the optimal lines from our series, which produce early xenografts and highlight the range of individual tumours from multiple mice in each of the key lines. This illustrates that the optimal line for consistent rapid tumour growth for drug and therapeutic screening appears to be IC1MET. The remarkable conservation of the pathological changes found in the primary tumours is shown in the photomicrographs. Particularly striking are the hyperkeratotic lesions from a field of actinic dysplasia (hence PM—premalignant). As the xenografts are in SCID mice, this clearly provides a permissive environment for tumour development, which allows the intrinsic pattern of tumour differentiation to develop. However, in our published studies of organotypical cultures using keratinocytes and fibroblasts from patients who develop cSCC due to the rare genetic disease recessive dystrophic epidermolysis bullosa (RDEB-type VII collagen deficiency), RDEB cancer-associated fibroblasts (CAFs) increased the adhesion and invasion of both tumour (including IC1) and non-tumour keratinocytes [32]. It would therefore be of interest to develop xenografts incorporating components of the tumour microenvironment (TME) such as CAFs, and also in mice with immune reconstitution using human immune cells in future experiments. Current xenograft models would be more suitable for testing targeted therapies rather than immune modulators.

### 3.3. Characterisation of cSCC Cell Lines for Future Experimentation

There have been a number of potential problems in the use of cSCC cell lines derived from tissue collections or by multiple partnering labs using those cell lines for experimentation. For example, analysis of 122 HNSCC lines (including three cSCC lines SRB1, SRB12, Colo16) authenticated only 85 unique cell lines by STR DNA profiling within this collection and highlighted misidentification and cross-contamination as common problems [6]. Details of STR DNA profiling of our cSCC cell line panel authenticated against the primary tumour of origin has therefore been presented here in order to provide researchers with an efficient method of verifying our cell lines [6]. Additionally, the morphology of the cell lines and the expected growth curves have been included for the assistance of new users (Figure 2).

### 3.4. Methylation Profiling

Perturbations in epigenetic regulation have been associated with the oncogenic process across a number of tumours, and many currently used anti-cancer medications target an epigenetic mechanism, such as DNA methylation inhibitors. DNA methylation profiling of cancer cell lines, often paired with methylation profiling of additional tissue samples, represents a method for identifying potential targets of pharmacological targeting and gaining further insight into tumour biology.

Our cell lines harbor epigenetic marks with biological functions linked to cSCC tumourigenesis, such as hypomethylation of RUNX1, the activation of which is linked to SCC formation in mice [33]. WNT5A associated with cSCC invasiveness was also hypomethylated in cSCC cell lines [34]. Tumour suppressor TP53 promoter was previously shown to be hypermethylated in cSCC, and we detected CpG hypermethylation of tumour protein p53 inducible protein 3, which is involved in p53-mediated cell death.

An unsupervised clustering analysis of genome-wide methylation data was able to distinguish cSCC-derived cell lines from cultured NHK and to separate cSCC cell lines based on the histology of the original tumour, with isogenic cell lines clustering closely together. Taken together, this shows that cultured cSCC cell lines maintain key epigenetic characteristics concordant with the original phenotype and retain certain critical epigenetic alterations inherent to the process of cSCC oncogenesis, and that cSCC lines may be utilised for rapid screening and identification of novel epigenetic marks and targets in cSCC.

### 3.5. Application of cSCC Cell Lines in Drug Screening

Cancer-derived cell lines have been widely used to develop anti-cancer drugs by high throughput screening and predictive modelling [35,36]. Although they tend to be developed from aggressive tumours and are therefore imperfect models alone for drug discovery programmes, they are not limited by resource or tumour size, a significant factor when using cSCC for patient-derived xenografts. Screening of the proteasome inhibitors bortezomib, ixazomib and carfilzomib, and ubiquitin inhibitors has been performed on normal keratinocytes and eight cSCC lines including IC1, IC1MET, MET1 and MET4 by assessing the effects on cell viability and cell death of long and short exposure to these clinically approved drugs. There were variations in cell line sensitivity/resistance [37]. Following an siRNA screen which identified the spliceosome as a potential target for cSCC therapy, small molecule inhibitors of the spliceosome targeting the splicing factor SF3B1/SF3b155 have also been studied on our cell lines (IC1, IC1MET, IC8, IC12, IC18, MET1 and MET4). They are more sensitive to pladienolide B than normal cells [38]. The effects of mitogen-activated protein kinase kinase (MEK) inhibition on cSCC responses were tested using two MEK inhibitors, trametinib and cobimetinib, in 10 cell lines (including IC1, T2, T3, T8), nine of which responded at high concentrations. However, this sensitivity was not correlated with the mutational status of RAS or epidermal growth factor receptor (EGFR) [27].

### 3.6. The Preclinical Pipeline

**Figure 6:**
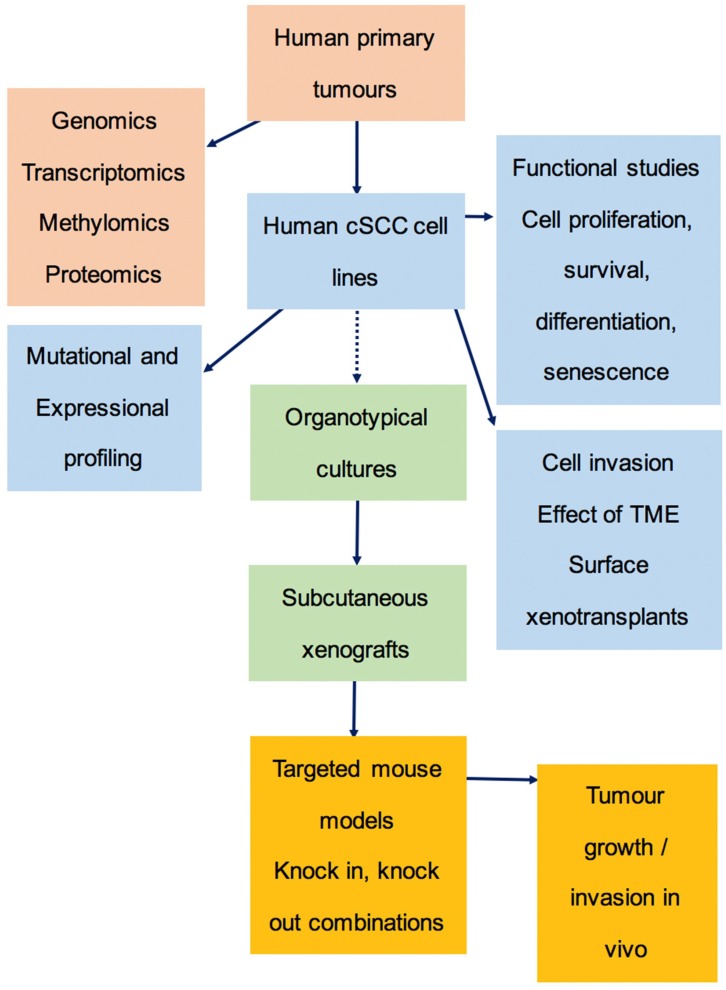
Preclinical pipeline. A pipeline diagram demonstrating the process of cSCC cell line establishment and characterisation, and potential preclinical investigations. TME; tumour microenvironment.

Animal models have been widely used to study skin carcinogenesis. Chemical carcinogenesis induced by tumour initiator and promotor-DMBA/TPA treatment has been the basis of a widely studied animal model of cSCC. These tumours differ from human disease as they develop predominantly papillomatous lesions. Although they have similar driver gene mutations in, for example *NOTCH* 1 and 2 [39], they bear much higher levels of *HRAS* mutation. In patients, lesions tend to progress from normal skin to premalignant actinic keratoses bearing dysplastic keratinocytes, through to invasive tumours. This morphology is better modelled in the solar-simulated ultraviolet radiation (SSUV) mouse, where chronic UV exposure of hairless mice produces keratotic lesions, which are phenotypically and genetically closer to the human tumours [40]. However, this requires very prolonged UV exposure, which limits the numbers of animals available. We have therefore developed a preclinical pipeline, which we believe has the power to identify relevant human carcinogenic pathways (Figure 6). Key to this is our human cSCC cell line panel used in organotypical cultures, together with subcutaneous and surface xenografts. We then confirm the findings in engineered mouse models as proof of principle for the human studies, as described in our publication on the role of TGFbeta receptors in squamous carcinogenesis [41].

### 3.7. Models for Metastasis

Key to understanding the high risk of metastasis of cSCC in OTRs [42] has been the ability to develop paired cell lines from both primary cSCC and lymph node metastasis, in both immunocompetent (IC1, IC1MET) and immunosuppressed (MET1/MET4) individuals [43]. By analyzing WES mutational data associated with branching from trunk to metastasis, we have constructed a phylogenetic tree (Figure 4), and this will be a starting point for further investigating key drivers of cSCC progression. There are few models of cSCC metastasis, but these cell lines could be used to screen for agents targeting metastatic disease. Regulatory requirements mandate that the animals bearing tumour xenografts have to be sacrificed once tumours reach a certain size, often before metastases can develop. However, this may be circumvented by systemic administration of tumour cells or by removing the primary tumour. These cSCC cell lines will be important for developing new models of metastasis.

## 4. Materials and Methods

### 4.1. Patient Samples

Clinically diagnosed cutaneous SCC lesions were surgically removed from patients attending dermatology clinics at our institution. Histopathologic diagnosis of all samples was confirmed by an experienced dermatopathologist. A 4–6 mm punch biopsy was placed into collection media comprising Dulbecco’s modified Eagle’s medium (DMEM, Life Technologies Ltd., Paisley, UK) supplemented with 5% fetal bovine serum (FBS, Biosera, Ringmer, UK), 100 units/mL penicillin, 100 µg/mL streptomycin and 2.5 µg/mL amphotericin B (all Life Technologies Ltd., Paisley, UK). Venous blood samples were drawn into ethylenediaminetetraacetic acid (EDTA) tubes, aliquoted into cryovials and stored at −80 °C. Ethical approval (REC Reference 08/S1401/69, 05 November 2008) for this study was obtained from the East London and City Health Authority local ethics committee and the study was conducted according to the Declaration of Helsinki Principles. All patients participating in the study provided written, informed consent.

### 4.2. Establishment of cSCC Cell Lines

Cell lines were established from cutaneous SCC tissue as described in Purdie et al. 2011 [44]. In brief, tumour biopsies were washed in PBS followed by 1 × versene (EDTA, Life Technologies Ltd., Paisley, UK) and transferred to a Petri dish containing 0.25% trypsin (Life Technologies Ltd., Paisley, UK) and then cut into small fragments 1–2 mm in size and incubated at 37 °C for an hour with intermittent agitation. The tissue was dissociated with needles and the suspension mixed with an equal volume DMEM supplemented with 10% FBS and passed through a 100 µm cell strainer. Cells were recovered by centrifugation, washed with PBS, resuspended in keratinocyte medium (a 3:1 V/V mixture of DMEM and Ham’s F12 (Life Technologies Ltd., Paisley, UK) supplemented with 10% FBS and a cocktail of mitogens: 0.4 µg/mL hydrocortisone, 10–10 M choleratoxin, 5 µg/mL transferrin, 2 × 10–11 M liothyronine, 5 µg/mL insulin, 10 ng/mL epidermal growth factor (EGF); all mitogens from Sigma-Aldrich, Poole, UK with the exception of cholera enterotoxin from Enzo Life Sciences Ltd., Exeter, UK and mouse EGF from AbD Serotec, Oxford, UK) and plated into a flask pre-seeded with mitotically inactivated Swiss 3T3 fibroblast feeder cells. Cultures were grown at 37 °C in 10% CO_2_. Upon reaching 80% confluence, cells were detached from the flask with trypsin/EDTA, washed in PBS and re-seeded at a 1:3 ratio. Cultures that had not senesced after 10 passages were considered to display extended lifespan and met the criteria for inclusion in this study.

### 4.3. 3-D Organotypic Cultures

Collagen/Matrigel gels were prepared by mixing on ice 3.5 volumes of collagen type I (Marathon Laboratory Supplies, London, UK) with 3.5 volumes of Matrigel^®^ (Becton-Dickinson, Oxford, UK), 1 volume 10× MEM and 1 volume FBS. The solution was equilibrated with 1 M NaOH and 1 volume DMEM/10% FBS containing normal human fibroblasts (NHF) at a concentration of 2 × 10^6^/mL added. Polyethylene terephthalate 0.4 μm inserts (Sarstedt, Leicester, UK) were placed into 24-well plates before 200 μL of the gel solution was added to each and left for 1 h at 37 °C and 5% CO_2_ to polymerise. Keratinocyte cells, in a media volume of 200 μL per well, were added at a density of 1 × 10^6^/mL. The cell suspension was added to the top of the gel and 400 μL of medium was added to the bottom of the well. Inserts were incubated at 37 °C at 5% CO_2_ for 24 h. After overnight incubation, the gels were lifted on steel grids with sufficient keratinocyte medium added to reach the undersurface of the gel and permit the epithelial layer to grow at the air–liquid interface. The gels were harvested after 7 days, fixed in paraformaldehyde and embedded in paraffin. Invasion was assessed by microscopic examination of 4 µm sections stained with hematoxylin and eosin (H&E).

### 4.4. Growth Curve and Cell Migration Assay

Cells were imaged and seeded for growth and migration assays at passages(p)27–28 for MET1 and MET4, p25 for IC8, p18–20 for IC18, MET2, IC1 and IC19, p15–16 for PM1 and T2, p11–12 for T1, T2, T10, T11 and IC1MET. Cells were seeded in 6-well plates at a density of 8 × 10^4^/well in keratinocyte medium at 37 °C. Cells were dissociated using 0.05% trypsin (Life Technologies Ltd., Paisley, UK) and counted on days 3, 5 and 7 post-seeding. A Transwell^®^ system incorporating a polycarbonate filter membrane (6.5 mm diameter, 8 µm pore size; Corning, Sigma-Aldrich, Poole, UK) was used to assess the rate of cell migration. Mitomycin C-treated keratinocytes (1 × 10^5^) were suspended in 100 µl of 0.1% bovine serum albumin (BSA) in DMEM/HamF12 (3:1 *v*/*v*) medium and seeded in the upper chamber of the Transwell^®^ insert. The lower chamber was filled with 600 µl of keratinocyte medium. Following 18 h of incubation at 37 °C, non-migrating cells on the upper surface of the filter were removed using a cotton swab. Cells that had migrated to the lower surface of the filter were stained with 1% Borax and 1% methylene blue before being lysed with a solution of 1% sodium dodecyl sulfate (SDS). Migration rate was determined by calculating the absorbance at 630 nm of the migrating cells as a proportion of the absorbance of the total number of cells seeded. The experiment was performed in triplicate a minimum of 2 times.

### 4.5. In Vivo Xenograft Experiments

Female SCID Balb/c mice or male CD1 nu/nu mice were subcutaneously injected in the right flank with 4 × 10^6^ or 5 × 10^6^ tumour cells respectively mixed with high-concentration Matrigel^®^ (Becton-Dickinson, Oxford, UK). Tumour volumes were measured twice a week with a caliper and calculated using the formula V = π4/3[(L + W)/4]3, where L is length and W is width. The time points which ended tumour sampling were determined by the point at which the tumours reached the size requiring sacrifice of the animal (800 mm^3^) or developed ulceration.

### 4.6. DNA Extraction and STR Profiling Analysis

DNA was extracted from cultured cells and blood using the Qiagen DNA Mini kit (Qiagen, Crawley, UK) according to the manufacturer’s instructions. Quantitation was performed using the double-stranded DNA-specific Qubit dsDNA BR assay kit in conjunction with the Qubit 2.0 fluorometer (both Life Technologies Ltd., Paisley, UK). Short tandem repeat (STR) genotyping was performed to authenticate the unique identity of each cell line. Sixteen loci distributed across the human genome were amplified using the AmpFLSTR Identifiler PCR Amplification Kit followed by capillary sequencing on the 3730xl DNA Analyzer (all reagents from Life Technologies Ltd., Paisley, UK). For SNP 6.0 analysis, DNA samples from cultured cells and paired non-tumour blood samples were hybridised to the microarrays according to the manufacturer’s protocols (Affymetrix, High Wycombe, UK). Exome sequencing was performed by Oxford Gene Technology (OGT), using Agilent SureSelect All Exon v5 (Agilent Technologies LDA UK Ltd, Cheshire, UK) for exome capture. Briefly, 1 µg DNA from each sample was used to prepare the sequencing library through shearing of the DNA followed by ligation of sequencing adaptors. Sequencing was performed on the Illumina HiSeq platform (Illumina, Inc., San Diego, CA, USA). Paired-end sequencing (2 × 100 bp) was carried out using HiSeq sequencing instruments.

### 4.7. Whole Exome Sequencing (WES) Data Processing and Analysis

The WES data generation and full analytical procedure has been described previously in detail (accession number GSE98780) [3].

### 4.8. Copy Number Analysis

Two independent approaches were applied to the copy number analysis of WES data. First, to generate single nucleotide polymorphism (SNP) and indel variant genotyping information, the tumour-normal pair was processed against the reference genome using the VarScan2 germline variants calling method. The minimum coverage for identified sites was 10 reads for both tumour and normal. Next, the logR and BAF (B-allele frequency) files were created based on the tumour-normal pair genotyping information, with the depth information normalised by dividing the depth of each variant by the median depth across all variants. The ASCAT R package [45] was then used to perform allele-specific copy number analysis to identify copy number alterations (CNA) and loss-of-heterozygosity (LOH) regions. The second approach was based on the numbers of reads aligned to each exon between the tumour and normal pair. VarScan2 copy number calling method was firstly applied. Raw copy number calls were adjusted for bases guanine and cytosine (GC) content and re-centred to 0 based on the modal logR value determined by kernel density estimates. Outliers were identified and adjusted using the data winsorsing procedure. The DNAcopy R Bioconductor package was then employed to identify joint segments of logR values using the circular binary segmentation (CBS) algorithm [46]. For SNP 6.0 microarray data, copy number analysis was performed using the aroma.affymetrix R package [47]. First, the CRMA v2 algorithm [48] was used for data pre-processing, normalisation and allele-specific copy number estimates. Paired parent-specific circular binary segmentation (Paired PSCBS) [49] was further performed on the tumour-normal pair to derive somatic CNAs and decrease-of-heterozygosity (DH). The ASCAT R package was also used for comparison purposes.

### 4.9. Phylogenetic Analysis

For two patients where multiple tumour cell lines were generated and sequenced, evolutionary trees were constructed on the basis of distance matrix among normal control and multiple cell line samples, using the neighbour-joining algorithm [50] implemented in the PHYLIP package (http://evolution.genetics.washington.edu/phylip.html). The non-silent mutations were used for this analysis. The mutations in the tree trunk were defined as “early” events, and the mutations in the branches after the tree split were “late” events.

### 4.10. Genome-Wide Methylation Profiling

A total of 6 cSCC lines characterised within this manuscript were used for the detection of differentially methylated genes. Three early-passage primary normal human keratinocytes derived from 3 different participants served as a normal control for comparison. DNA was extracted as described above and bisulfite modification was carried out as the following: 500–1000 ng of DNA yield from each sample was modified with bisulfite using the EZ DNA Methylation Kit (Zymo, Research, CA, USA) according to the manufacturer’s instructions. Methylation detection was performed using the Illumina Infinium HumanMethylation27 BeadChip. This platform detects the methylation status of 27,578 CpG sites spread across 14,495 genes by sequencing-based genotyping of bisulfite-converted DNA. Eventual methylation scores (denoted “beta-value”) were generated for each site with BeadStudio software (Illumina, Inc., San Diego, CA, USA) and raw background-corrected values were used for further analysis. The methylation assay was performed according to the manufacturer’s instructions. Briefly, bisulfite-converted DNA was amplified, fragmented and hybridised to the chip arrays, followed by imaging with the Illumina BeadArray reader. Image processing and intensity data extraction were performed according to Illumina’s instructions. All analyses were carried out using Bioconductor and R.

## 5. Conclusions

We present a definitive analysis of a unique set of cSCC lines that can be used as a model of skin squamous carcinogenesis for in vivo and in vitro studies. We provide details of their extensive characterisation, including clinical, phenotypic, genotypic, in vitro and in vivo properties. In addition to the research reported to date using these cell lines, we anticipate that this unique resource will continue to provide researchers with a powerful preclinical model for future investigations of the molecular drivers of cSCC progression, the identification of novel therapeutic targets and, ultimately, the development of more effective treatment strategies for this common skin cancer.

## Figures and Tables

**Table 1:** Details of established cell lines, patient characteristics, immune therapies, histopathological status, and identification of in vivo and in vitro tests.

Cell line	Tumour	Immune Status	Immune Therapy ^1^	Site	SCC Histology	Age	Sex	Germline DNA ^2^	Organotypic Invasion	Transwell Migration	Tumorigenicity ^3^
PM1 ^#^	pre-malignant lesion	RT	A,P	scalp	dysplastic	45	M	dermal fibroblasts	ND	ND	0/10 ^4^, 6/6
MET1 ^#^	primary	RT	A,P	dorsum left hand	MD	45	M	dermal fibroblasts	ND	40–100%	5/10 ^4^, 6/6
MET2 ^#^	recurrence of MET1	RT	A,P	dorsum left hand	MD	45	M	dermal fibroblasts	ND	40–50%	7/10 ^4^
MET4 ^#^	metastasis of MET 1/2	RT	A,P	left axillary lymph node	metastasis	46	M	dermal fibroblasts	ND	40–60%	5/10 ^4^; 6/6
SCC T9 ^#^	unrelated primary of MET patient ^#^	RT	A,P	right hand	WD	45	M	dermal fibroblasts	ND	ND	ND
SCC IC1	primary	IC	N/A	right temple	MD	77	M	lymphocytes		30–50%	14/14 ^5^; 6/6
SCC IC1MET	metastasis of IC1	IC	N/A	right preauricular lymph node	metastasis	77	M	lymphocytes	ND	10–50%	6/6
SCC IC8	primary	IC (PUVA)	N/A	buttock	PD	51	F	lymphocytes		20–25%	ND
SCC IC12	primary	IC	N/A	left calf	MD-PD	87	F	lymphocytes	ND	ND	ND
SCC IC18	primary	IC	N/A	right ear	MD	81	M	lymphocytes	ND	ND	ND
SCC IC19	primary	IC	N/A	scalp	MD	81	M	lymphocytes	ND	20–50%	ND
SCC T1	primary	RT	C, P	forearm	WD	61	M	N/A	ND	30–50%	0/4 ^5^
SCC T2	primary	CT	A, C, P	hand	WD	66	M	lymphocytes	ND	ND	2/3 ^5^
SCC T8	primary	RT	C, P	ear	PD	67	M	lymphocytes	ND	ND	3/4 ^5^
SCC T10	primary	RT	A *, C, P	left shin	MD	60	M	lymphocytes	ND	ND	ND
SCC T11	primary	RT	A *, C, P	back	PD	48	M	lymphocytes	ND	ND	ND

^#^*all cells derived from the same patient*; *IC = Immuno-competent, RT = renal transplant, PUVA= psoralen + ultraviolet A, CT = cardiac transplant, ND= not determined*; ^1^ Patients received imunosuppressive therapy as follows: A, azathioprine; C, Cyclosporine A; M, mycophenolate mofetil (MMF); P, prednisolone; *, patient had stopped treatment by the time of lesional biopsy; ^2^ Whole exome sequencing was only performed on those cell lines where germline DNA was available; ^3^ Proportion of mice (female SCID Balb/c unless otherwise indicated) with detectable tumours after bolus subcutaneous injection of cells; ^4^ Data from earlier xenograft experiments performed in male CD1 nu/nu mice using cells at culture passage pp. 8–15 (MET lines) or p > 40 (PM1) (previously published in Proby et al 2000); ^5^ Data previously published in Watt et al 2011 using cells at culture passage pp. 18–24.

**Table 2:** Summary of short tandem repeat (STR) genotyping gives a characteristic genotype for the identification of individual cell lines.

Cell Line	AMEL	CSF1PO	D13S317	D16S539	D18S51	D19S433	D21S11	D2S1338	D3S1358	D5S818	D7S820	D8S1179	FGA	TH01	TPOX	vWA
SCC IC1	X, Y	12, 14	9, 13	9, 13	15, 17	15, 16.2	29, 30	19, 25	14, 17	11, 13	8, 11	13, 13	21, 24	9.3, 9.3	8, 8	16, 16
SCC IC1MET	X, Y	12, 14	9, 13	9, 13	15, 17	15, 16.2	29, 30	19, 25	14, 17	11, 13	8, 11	13, 13	21, 24	9.3, 9.3	8, 8	16, 16
PM1	X, Y	9, 13	8, 12	11, 13	18, 19	14, 14	30.2, 33.2	17, 21	14, 14	11, 12	7, 8	12, 15	24, 24	8, 9.3	9, 11	18, 19
MET1	X, Y	13, 13	8, 12	11, 13	18, 19	14, 14	30.2, 33.2	17, 21	16, 16	11, 12	7, 8	12, 15	24, 24	9.3, 9.3	9, 11	18, 19
MET2	X, Y	13, 13	8, 12	11, 13	18, 19	14, 14	30.2, 33.2	17, 21	16, 16	11, 12	7, 8	12, 15	24, 24	9.3, 9.3	9, 11	18, 19
MET4	X, Y	13, 13	8, 12	11, 13	18, 19	14, 14	30.2, 33.2	17, 21	16, 16	11, 12	7, 8	12, 15	24, 24	9.3, 9.3	9, 11	18, 19
SCC T9	X, Y	9, 13	8, 12	11, 13	18, 19	14, 14	30.2, 33.2	17, 21	14, 16	11, 12	7, 8	12, 15	24, 24	8, 9.3	9, 11	18, 19
SCC IC8	X, X	12, 13	11, 11	11, 12	13, 18	13.1, 14	29, 29	17, 21	16, 16	13, 13	8, 12	16, 16	19, 23	9, 9	8, 8	15, 16
SCC IC12	X, X	12, 12	11, 12	12, 13	15, 16	13.1, 14	28, 31.2	17, 17	15, 15	12, 13	10, 11	11, 12	22, 24	6, 10	10, 11	17, 19
SCC IC18	X, X	11, 11	10, 11	9, 13	12, 12	13.1, 15	28, 32.2	17, 18	16, 17	13, 13	9, 11	8, 12	21, 21	6, 8	9, 9	14, 17
SCC IC19	X, X	11, 11	10, 12	9, 12	11, 18	13.1, 15.2	32.2, 32.2	19, 21	15, 15	12, 12	9, 12	13, 15	20, 22	6, 7	11, 11	16, 17
SCC T1	X, Y	12, 12	9, 12	12, 13	12, 14	14, 15	30, 32.2	18, 20	17, 17	12, 13	9, 10	13, 13	21, 24	9, 9.3	9, 12	14, 16
SCC T2	X, Y	10, 10	11, 14	10, 11	12, 13	14, 16	28, 31	17, 20	15, 15	11, 12	ND	13, 15	22, 22	6, 6	8, 9	15, 18
SCC T8	X, X	10, 10	13, 13	11, 12	12, 13	14, 15	31.2, 32.2	25, 25	14, 15	12, 12	11, 12	13, 13	21, 23	8, 8	11, 11	14, 19
SCC T10	X, Y	10, 11	8, 9	9, 10	13, 19	15, 15	29, 30	22, 24	16, 17	11, 11	ND	10, 15	24, 25	9, 9	8, 11	14, 17
SCC T11	X, Y	10, 12	11, 12	12, 13	11, 13	12, 14	29, 30	17, 19	14, 16	11, 11	7, 12	11, 15	19, 20	6, 9.3	8, 8	18, 18

## Abbreviations

cSCC	cutaneous squamous cell carcinoma
OTR	organ transplant recipients
SCID	severe combined immune deficiency
STR	short tandem repeat
HNSCC	head and neck squamous cell carcinoma
ATCC	American Type Culture Collection
WES	whole exome sequencing
CNA	copy number alterations
DAVID	Database for Annotation, Visualization and Integrated Discovery
FDR	False discovery rate
NHK	normal human keratinocyte
ECACC	European Collection of Authenticated Cell Cultures
DSMZ	German Collection of Microorganisms and Cell Cultures GmbH
HPV	human papillomavirus
CAFs	cancer-associated fibroblasts
RDEB	recessive dystrophic epidermolysis bullosa
TME	tumour microenvironment
MEK	mitogen-activated protein kinase kinase
EGFR	epidermal growth factor receptor
SSUV	solar-simulated ultraviolet radiation
EDTA	ethylenediaminetetraacetic acid
EGF	epidermal growth factor
H&E	hematoxylin and eosin
BSA	bovine serum albumin
SDS	sodium dodecyl sulfate
NHF	normal human fibroblasts
SNP	single nucleotide polymorphism
LOH	loss of heterozygosity
CBS	circular binary segmentation

## Appendix A

**Table A1:** Summary of previous publications using recently fully characterised cSCC lines.

Reference	Cell Lines Utilised (Nomenclature Changes) [N.B. SCC Prefix Sometimes Included]	Experimental Overview
[7]	PM1, MET1, MET2, MET4	PM1 and additional isogenic premalignant cells retain features of normal keratinocytes compared to MET1-4 cells, which displayed reduced growth requirements, abnormal differentiation, aberrant K18 expression and tumorigenicity in vivo. HPV is not necessary for immortal phenotype maintenance.
[43]	MET1, MET2, MET3, MET4	Genetic analysis of MET series.
[51]	PM1, MET1, MET4	The oncogenic receptor tyrosine kinase Axl plays a role in driving cSCC; it is upregulated in MET1 and MET4 compared to PM1.
[52]	MET1, SCCIC1	Type VII collagen knockdown increases cell migration and invasion in MET1 and SCCIC1 cells. Knockdown also leads to the disorganisation of epithelial differentiation and the promotion of epithelial to mesenchymal transition.
[53]	MET1, MET4	The flavonoid luteolin inhibited UVB-induced inflammation and apoptosis in normal human keratinocytes. Exposure to luteolin did not increase cSCC line UVB resistance.
[54]	PM1, MET1, MET4	Sensitisation of cSCC cells to cisplatin-induced cell death is achieved by the induction of apoptosis after Akt inhibition and can be increased with an Akt- and autophagy-inhibitor combination.
[55]	PM1, MET1	The activated DNA damage response protein kinase ATM (pATM) is located in the perinucleus and, focally, in the cytoplasm of PM1 and MET1 cSCC cells. UVB-irradiation resulted in a degree of nuclear localisation in premalignant PM1 but not MET1 cells. DNA damage response proteins are upregulated in early carcinogenesis.
[56]	MET1, SCCIC1	Axl knockdown in MET1 sensitises cells to UV-induced apoptosis via increased activation of pro-apoptotic protein Bad, altered conformation of pro-apoptotic proteins Bax and Bak, cytosolic cytochrome c release and caspase activation. Expression levels upon Axl knockdown were confirmed in SCCIC1 cells. UV-induced apoptosis was via the mitochondrial-mediated pathway. Axl confers cSCC resistance to apoptosis.
[57]	SCCT1, SCCT2, SCCT8, SCCIC1, SCCIC8, SCCIC12	NOTCH1 and NOTCH2 mutations found in ~75% of cSCCs lines illustrating central roles in the disruption of microenvironmental communication in cSCC progression.
[58]	SCCT1, SCCT2, SCCT8, SCCIC1	The overexpressed cSCC tumour cell-survival genes, PLK1 and C20orf20, when inhibited by siRNA-mediated knockdown, induced apoptosis in vitro and tumour growth reduction in vivo.
[59]	MET1, MET4	The retinoid signalling molecule TRIM16 is reduced during progression from normal to actinic keratosis and cSCC. In cSCC TRIM16, cytoplasmic and nuclear expression is lower than normal lines due to reduced TRIM16 stability. In cSCC TRIM16, bound and downregulated E2F1 are required for replication. In normal cells, retinoids increase nuclear TRIM16 but not in retinoid-resistant cSCC. Overexpression of TRIM16 in cSCC reduced migration. TRIM16 plays a role in cSCC development and retinoid sensitivity.
[60]	MET1, MET4	The flavonoid luteolin induced caspase-dependent cell death in cSCC. No cytotoxicity occurred in normal cell lines. Luteolin-induced apoptosis was accompanied by Akt signalling inhibition. Sensitivity decreased with tumour progression, with MET1 more sensitive to luteolin than MET4. Luteolin stimulates autophagy in MET4 cells. In blocking autophagy, luteolin-induced apoptosis in MET4 was enhanced.
[32]	SCCIC1	Expression of full-length type VII collagen in RDEB fibroblasts retards SCCIC1 growth and invasion compared to growth and invasion facilitated by RDEB fibroblasts expressing empty vectors. Matrix composition in RDEB patients provides a tumour permissive environment, with type VII collagen directly regulating cancer-associated fibroblast protein secretion and cSCC progression.
[61]	PM1, MET1, MET4	Sensitivity to apoptosis induced by UVB or oxidative stress differs between isogenic cell lines compared to cisplatin-induced apoptosis. MET1 was most sensitive to UVB- and oxidative stress-mediated apoptosis, with PM1 being the most resistant to apoptosis induction.
[62]	MET1, SCCIC1	Axl knockdown in MET1 cells leads to ALDH1 cancer stem marker downregulation, CTGF, TWIST and VIM EMT marker downregulation, and E-cadherin upregulation. Subpopulations of MET1 cells expressing higher levels of cancer stem marker CD44 have correspondingly higher levels of Axl expression. Morphological and cell–cell interaction changes occur with Axl knockdown. Chemotherapeutic drug-induced cell death was inhibited by Axl expression.
[63]	MET1, MET2, MET4, SCCT8, SCCIC8, SCCIC18, SCCIC19	The actin regulatory scaffold protein Eps8 is elevated in cSCC compared to normal skin. There were no differences in Eps8 levels based on cSCC progression or immune status. Eps8 in cSCC forms as a complex with focal adhesion kinase (FAK) at focal adhesions, promoting actin-associated cancer phenotypes, including direction sensing and invasive migration. In the absence of FAK, Eps8 is co-recruited with active Src. This provides a role for Eps8 as a mediator of intracellular kinase fate in cSCC.
[64]	SCCT1, SCCT2, SCCT8, SCCIC1, MET1, SCCIC1MET, MET4	The SLCO1B3 gene, encoding the anion transporter frequently-mutated in cancer, OATP1B3, is upregulated in RDEB cSCC and UV-induced cSCC compared to normal keratinocytes. With the exception of SCCT8, all cSCC cell lines showed lower expression of SLCO1B3 compared to RDEB cSCC keratinocytes. Compared to normal keratinocytes, OATP1B3 is expressed in cSCC keratinocytes; however, membrane-localised expression is only readily detected in 3D cultures, mouse xenografts or in OATP1B3-overexpressing 2D cultures. COL7A1 knockdown increases SLCO1B3 expression in SCCT8 cells. SLCO1B3 expression was also regulated by COL7A1 and ELMO2 in cSCC cells.
[65]	MET1, MET2, MET4	Flightless I is elevated in cSCC lines compared to HaCaT cell line controls. There was no significant difference in expression between isogenic lines MET1, MET2 and MET4.
[66]	SCCIC1	Type VII collagen knockdown in SCCIC1 cells reduces cell–cell adhesion, increases blood vessel formation by increased vascular endothelial growth factor (VEGF) secretion, increases invasion, and reduces differentiation in cellular, organotypic and xenograft studies.
[41]	SCCIC1, SCCIC8, SCCIC12, SCCIC18, SCCIC19, SCCT1, SCCT2, SCCT8, PM1, MET1, MET4, SCCT9, SCCT10, SCCT11	Identification of frequent TGFBR1/2 mutations in human vemurafenib-induced skin lesions and in sporadic cSCC. LGR5+ stem cells may act as origins for cSCC, driven by RAS/RAF/MAPK pathway hyperactivation or TP53 mutation (coupled with reduction in TGFb signalling). The TGFBR2-mutant cell lines SCCIC8 and SCCIC12 failed to respond to exogenous TGFb stimulation.
[27]	SCCT2, SCCT8, SCCIC1	MEK inhibition results in senescence, but not apoptosis. Sensitivity to MEK inhibition was seen across all lines at high concentration, low concentration-response between lines was heterogenous. MEK inhibition may be a basis for molecularly targeted cSCC chemoprevention and therapy.
[67]	SCCIC18	Activated SMAD2/3 was significantly reduced in perilesional cSCC and, to a greater extent, lesional cSCC tissue. Increased tumour thickness inversely correlates with phospho-SMAD presence. Lines were used for IHC antibody validation.
[37]	MET1 (**SCCT**), MET4 (**SCCTMet**), SCCIC1, SCCIC1Met	Continuous treatment with the ubiquitin E1 inhibitor MLN7243 selectively killed MET1 and SCC IC1MET cells. The latter cells were resistant to the inhibitor bortezomib. SCCIC1 cells were susceptible to bortezomib pulses but highly resistant to MLN7243. Bortezomib-resistant cells can be sensitive to MLN7243. Generally, pulse proteasome inhibitor exposure kills cSCC cells more effectively than extended exposure. C-MYC-dependent pro-apoptotic protein NOXA upregulation confers inhibitor susceptibility for rapid BAK-dependent death.
[68]	T1, T8, MET1, MET4	Early stage T1 demonstrates the greatest proliferative response to IL-22 treatment. cSCC proliferation was inhibited by JAK1/2 inhibitor ruxolitinib in vitro with tumour size reduction in vivo; however, this was demonstrated in the A431 cSCC epidermoid carcinoma line only and not in T1, T8, MET1 or MET4 lines.
[3]	All lines (nomenclature consistent)	Whole exome sequencing analysis confirmed COSMIC mutation signature 7 was present in all lines. Signature 32 was present in eight lines (four patients), all immunosuppressed and receiving azathioprine. PM1, MET1/2/4 and T9 (all isogenic lines) showed similar mutational signatures. MET4 had additional signature 26 associated with defective DNA mismatch repair. Altered mismatch repair genes are found in all cSCC cell lines, but none correlate with signature 26.
[38]	MET1 (**SCCT**), MET4 (**SCCTMet**), SCCT1, SCCT2, SCCT8, SCCT10, SCCT11, SCCIC1, SCCIC1Met, SCCIC8, SCCIC12, SCCIC18	Spliceosome suppression has potential as a cSCC treatment. Endogenous C-MYC expression in cSCC cells plays a role in conferring spliceosome inhibition-sensitivity and can act as a determinant of cSCC sensitivity to spliceosome targeting. Mutant p53 depletion does not attenuate spliceosome-inhibition-induced cell death in cSCC cells.
[69]	MET1, IC1	Dual-action lysine specific demethylase 1- and histone deacetylase- inhibitor, coined Corin, exhibits anti-proliferative activity in cSCC lines, compared to mono-functional inhibitors alone.
[70]	MET1, MET4, IC8	The HDAC-inhibitor MS-275 potently inhibits the proliferation of MET4, MET1 and IC8 cells. MS-275 also induced expression of p21 in cSCC cells.
